# Electronic structure of vertically coupled quantum dot-ring heterostructures under applied electromagnetic probes. A finite-element approach

**DOI:** 10.1038/s41598-021-83583-5

**Published:** 2021-02-17

**Authors:** M. E. Mora-Ramos, J. A. Vinasco, D. Laroze, A. Radu, R. L. Restrepo, Christian Heyn, V. Tulupenko, Nguyen N. Hieu, Huynh V. Phuc, J. H. Ojeda, A. L. Morales, C. A. Duque

**Affiliations:** 1 Centro de Investigación en Ciencias-IICBA, Universidad Autónoma del Estado de Morelos, Av. Universidad 1001, CP 62209 Cuernavaca, Morelos, Mexico; 2 Instituto de Alta Investigación, CEDENNA, Universidad de Tarapacá, Casilla 7D, Arica, Chile; 3 Department of Physics, University Politehnica of Bucharest, 313 Splaiul Independenţei, Bucharest, 060042 Romania; 4 Universidad EIA, CP 055428 Envigado, Colombia; 5 Center for Hybrid Nanostructures (CHyN), University of Hamburg, Luruper Chaussee 149, 22761 Hamburg, Germany; 6 Donbass State Engineering Academy, Academichna str. 62, Kramatorsk, 84313 Ukraine; 7 Institute of Research and Development, Duy Tan University, Da Nang, 550000 Viet Nam; 8 Faculty of Natural Sciences, Duy Tan University, Da Nang, 550000 Viet Nam; 9 Division of Theoretical Physics, Dong Thap University, Cao Lanh, 870000 Viet Nam; 10 Grupo de Física de Materiales, Facultad de Ciencias, Universidad Pedagógica y Tecnológica de Colombia, Tunja, Boyacá Colombia; 11 Grupo de Materia Condensada-UdeA, Instituto de Física, Facultad de Ciencias Exactas y Naturales, Universidad de Antioquia UdeA, Calle 70 No. 52-21, Medellín, Colombia

**Keywords:** Electronic properties and materials, Quantum dots

## Abstract

We theoretically investigate the electron and hole states in a semiconductor quantum dot-quantum ring coupled structure, inspired by the recent experimental report by Elborg and collaborators (2017). The finite element method constitutes the numerical technique used to solve the three-dimensional effective mass equation within the parabolic band approximation, including the effects of externally applied electric and magnetic fields. Initially, the features of conduction electron states in the proposed system appear discussed in detail, under different geometrical configurations and values of the intensity of the aforementioned electromagnetic probes. In the second part, the properties of an electron-hole pair confined within the very kind of structure reported in the reference above are investigated via a model that tries to reproduce as close as possible the developed profile. In accordance, we report on the energies of confined electron and hole, affected by the influence of an external electric field, revealing the possibility of field-induced separate spatial localization, which may result in an indirect exciton configuration. In relation with this fact, we present a preliminary analysis of such phenomenon via the calculation of the Coulomb integral.

## Introduction

The research on quantum nanostructures has, as one of its highest peaks, the study of the physical properties of quasi-zero-dimensional systems such as quantum dots and quantum rings. The concept of semiconductor microcrystallites, as they were first named^1–5^, evolved to the term “quantum dot” (QD) in the work of Reed et al.^6^, and continued to be broadly investigated until present^7–9^, with significant technological applications in the fields of electronics and optoelectronics (see, particularly, the recent report by Won and collaborators^10^). The physics of QDs has been extensively explored, as reflects in a number of monographs and compilations published through the years^11–15^.

In the case of semiconductor quantum rings (QRs) of nanoscopic size we can mention, as a relevant starting point event, the discovery of the self-organized formation of QRs by García et al.^16^. The practical realization of this class of structures allowed a unique playground for a number of novel properties related with topological effects. In particular, the optical response associated to the features of the energy spectrum in this kind of systems was early investigated^17–19^.

A comprehensive compilation of many diverse works on the physics of QRs appears in the book of Fomin^20^. There, as well as in^21^, the reader will be able to find a large number of references on the subject. For instance, theoretical results on electronic, magnetic and optical properties of QRs are reported in^22^, whereas features of these nanosystems in electromagnetic fields are discussed in^23^. It is known that the influence of a magnetic field on a charged particle confined in a QR leads to the so-called Aharonov–Bohm effect^24^. Such phenomenon characterizes by the presence of persistent currents and oscillations of the particle ground state energy. Besides, magnetic field-related energy structure and magneto-optical transitions in QRs were reported in^25,26^. Excitons in QRs under magnetic field effects were investigated in^27–30^. A numerical modeling of InAs/GaAs QRs was presented in Ref.^31^, whilst the investigation of the electronic and intraband optical properties of GaAs-based ring structures under intense laser field appeared in^32,33^. Laterally applied electric field and hydrostatic pressure effects were studied in^34^. A very recent report on impurity-related effects is the one published by El-Bakkari and coworkers^35^. Studies on coupled QR systems have also appeared in the literature of recent years. In this sense, it is possible to mention the work by Wu and collaborators^36^ as well as those by Cattapan and Lotti^37^ and Escorcia et al.^38^ in laterally aligned QR systems. Electronic and exciton properties in concentric double rings have been the subject of research in^39–41^. Vertically stacked quantum rings have been considered, for example, in the works of Refs.^42–44^. QR-based systems may find prospective applications in the fields of optoelectronics (in the form of light emitters and lasers) and photonics as well as in spintronics and quantum information technologies^20^.

The semiconductor quantum dot-quantum ring (QDR) complex, in which charge carriers undergo the combined influence of -coupled- QD- and QR-type quantum confinement, has also been a subject of study^45–58^. Among these examples, there are several works devoted to fabricating QDR systems^46,47,50,54,56,58^. It is worth to highlight as well the very recent investigation by Heyn et al. in which a new kind of nanostructure related with the phenomenon of field-induced quantum dot to ring transformation in GaAs cone-shell quantum structures has been put forward^58^.

The interest of the present work is directly motivated by the research on self-assembled, vertically aligned, quantum ring-dot structures performed by Elborg and collaborators^54^. there, by making use of multiple droplet epitaxy (DE) technique, the QDRs were obtained by depositing GaAs QRs in a first droplet epitaxy process. These QRs were subsequently covered by a thin AlGaAs barrier and, in a second DE process, Ga droplets were grown, positioned either at the center indentation of the ring or attached to its edge. These droplets crystalized into GaAs QDs under a flux of As atoms. According to the authors, the designed growth technology would allow for selectively tuning the geometry of the dot-ring complex, which makes it attractive to possible quantum information applications.

**Figure 1:**
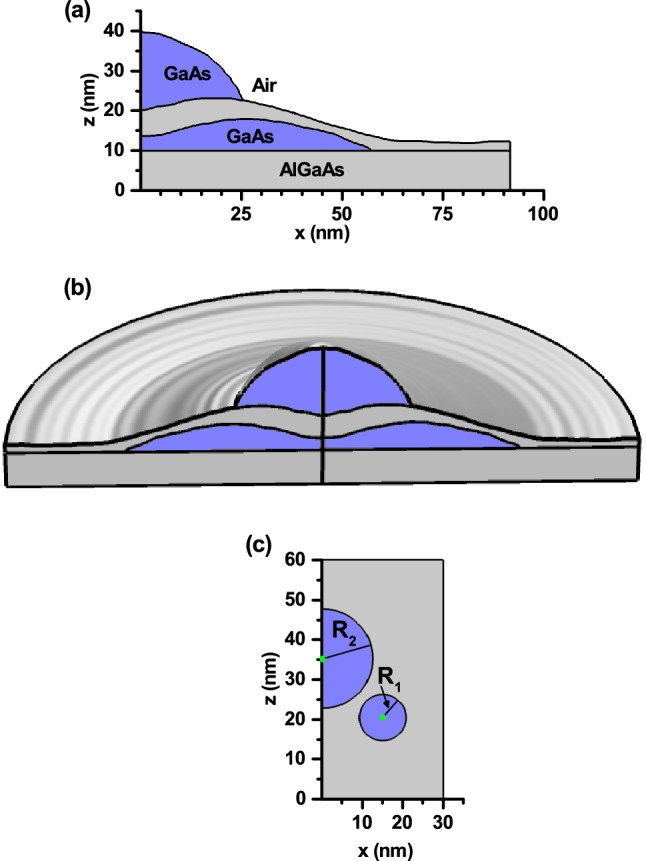
Schematic representation of a GaAs quantum dot-quantum ring system (blue color) embedded in a matrix of Al$_{0.28}$Ga$_{0.72}$As (gray color) and air^54^. The lateral building of the system (**a**) and a cut view of the system, after rotation of plot (**a**) around *z*-axis (**b**). In (**c**) is depicted the dot-ring model that will be used to simulate the real structure depicted in (**b**).

The purpose here is to carry out a theoretical study of the confined states of electrons and holes—and their electrostatic coupling—in the kind of system put forward in Ref.^54^, within the framework of the envelope function and effective mass approximations. Actually, the study primarily focuses on a simpler structure consisting in a coupled spherical quantum dot-cylindrical quantum ring; in order to compare with the more complex experimental structure which consist on a quasi-spherical dot and quasi-cylindrical ring.

The DE-fabricated QDR complex reported in^54^ consists of a strain-free QD located above a QR, both made of GaAs and surrounded by Al$_{0.28}$Ga$_{0.72}$As. In Fig. 1a,b we present the QDR model extracted from the above mentioned reference. Fig. 1a contains the plot of half of the $y=0$ plane projection of the structure, corresponding to $x\ge 0$. On the other hand, in Fig. 1b we present the cut view of the resulting QDR 3D model, obtained after rotating Fig. 1a around the *z*-axis. Fig. 1b clearly shows that the QD height is at least twice the maximum QR height Besides, the maximum QD radius reaches the same order of magnitude as the central radius of the annular region. These relative dimensions between the QR and the QD will have important effects on the localization of electrons and holes in the absence of external fields. Additionally, Fig. 1c presents the quantum dot-ring geometrical model that will be used to simulate the real structure.

**Figure 2:**
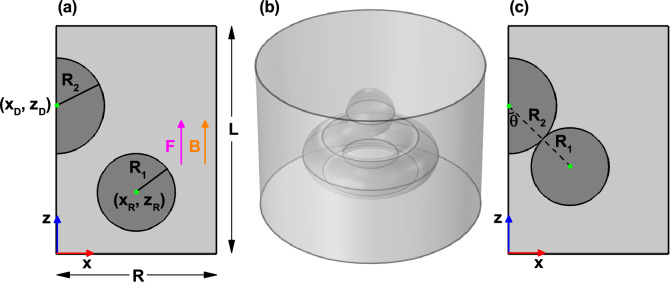
Schematic representation of the GaAs quantum ring and quantum dot system embedded in a matrix of Al$_{0.28}$Ga$_{0.72}$As. The half of the $y=0$ projection is depicted in panel (**a**), where $R_1$ and $R_2$ represent the ring and dot radii, respectively. The points $(x_R,z_R)$ and $(x_D,z_D)$ correspond to the coordinates of the ring and dot projections, respectively. The parameters $R=70$ nm and $L=100$ nm represent the size of the rectangle in which upper, lower, and right-hand sides Dirichlet conditions are imposed. *F* and *B* are the vertically applied electric and magnetic field, respectively. (**b**) 3D view of the system, after rotating the figure (**a**) around *z*-axis. (**c**) ring and dot projections when in touch. The position of the ring with respect to the dot is defined via the $\theta$-angle. Reference frame is localized at the left-bottom corner of the rectangle.

This work will also explore the possibility of using externally applied electromagnetic probes in order to identify suitable tools for modifying the spectrum of carrier states, together with changes in the geometry of the structure. These probes are static electric and magnetic fields, with suitable spatial orientations and their respective intensities varying within given -practically achievable- intervals. Being a mathematical problem that implies solving the 3D effective mass Schrödinger partial differential equation (SPDE) in the rather complex doubly-connected QR geometry, different methods have been proposed in the literature. For some particular models of the confining potential, the matrix diagonalization method has been the technique of choice^32–34,51,52,55,57^. Nevertheless, other authors have considered direct numerical schemes for the solution of SPDE, such as the finite difference approach^45^, discretization together with Lanczös diagonalization^53^, and the finite element method (FEM)^31^. From this variety of numerical approaches, we have chosen to use the FEM to obtain the solution for confined carrier states in a QDR prototype inspired in the structure developed in the above mentioned work (see schematic representation in Fig. 2). Our theoretical investigation also assumes the possibility of including the coupling of electrons and holes through Coulombic interaction. The results will be presented as functions of the geometric setup of the system as well as of the electric and magnetic field intensities.

The article has the following organization: “Theoretical model” contains details of the theoretical model employed. In “Results and discussion” the obtained numerical results are analyzed and discussed. “Conclusion” is devoted to present the corresponding conclusions.

## Theoretical model

The calculations were carried out for an electron and a hole, both confined within a GaAs complex formed by a quantum ring and a quantum dot (hereafter labeled as QDR). The radius of the QR transversal section is $R_1$, whereas $R_2$ corresponds to the dot radius as illustrated in Fig. 2a. In the same figure, $(x_R,z_R)$ and $(x_D,z_D)$ correspond to the coordinates of the ring and dot $y=0$ projections, respectively. The Fig. 2b is a 3D view of the QDR configuration, which is reached by rotation of Fig. 2a around the *z*-axis. In the model, we are assuming that the QDR is embedded inside a semiconducting Al$_{0.28}$Ga$_{0.72}$As matrix. In Fig. 2c we present the plot of ring and dot regions when they are in touch. The ring position, with respect to the dot, is defined via the $\theta$-angle. In this case, the dot coordinates are $(0,z_D)$, whereas the ring coordinates are $x_R=(R_1+R_2)\,\sin \theta$ and $z_R=z_D-(R_1+R_2)\,\cos \theta$.

In order to calculate the spectra of carriers in the QDR system, we use the effective mass theory and parabolic band approximation to write the main equation

1 $$\begin{aligned} \left\{ \frac{1}{2m_{W,B}^{*}}\left( -i\hbar \nabla -q\mathbf {A}_m \right) ^2 \right\} \psi (x,y,z)+\left[ V(x,y,z)-qF\left( z-z_0 \right) \right] \psi (x,y,z)=E\,\psi (x,y,z), \end{aligned}$$

where $\mathbf {A}_m=-\frac{B}{2}y\mathbf {i}+\frac{B}{2}x\mathbf {j}$ is the magnetic vector potential, $\mathbf {B}$ is the applied magnetic field, and $m_{W,B}^{*}$ is the effective mass. The sub-index *W* indicates the QDR region, and sub-index *B* labels for the Al$_{0.28}$Ga$_{0.72}$As potential barrier. The charge parameter is $q=-e, e$ for electron and hole respectively, while *V*(*x*, *y*, *z*) is the confinement potential, and *F* is the value of applied electric field, oriented along the $z-$direction. Here, $z_0$ is a parameter which will be tuned in order to accelerate the convergence of the calculations.

As depicted in Fig. 2, the system fulfills the azimuthal symmetry. Hence, using cylindrical coordinates, Eq. (1) can be split into two independent equations, one of them corresponding to the uncoupled azimuthal motion -depending on the angular variable $\varphi$- with magnetic quantum number *l*. Accordingly, the solution of Eq. (1) can be written as $\psi (\rho ,\varphi ,z)=R(\rho ,z)e^{il\varphi }$ (with *l* an integer number). The $R(\rho ,z)$ component is calculated from Eq. (2)

2 $$\begin{aligned} \left\{ -\frac{\hbar ^2}{2m_{W,B}^{*}}\nabla ^2 +V_s+V_m-qF\left( z-z_0 \right) \right\} R(\rho ,z)=E\,R(\rho ,z), \end{aligned}$$

where $V_s$ is the structural potential profile and $V_m$ contains the magnetic field components. They are respectively given by

3 $$\begin{aligned} V_s=\frac{\hbar ^2}{2m_{W,B}^{*}}\left( \frac{l}{\rho } \right) ^2+V(\rho ,z), \end{aligned}$$

with $V(\rho ,z)=0$ inside of the QRD system and $V_0$ outside, and

4 $$\begin{aligned} V_m=-\frac{q\hbar }{2m_{W,B}^{*}}Bl+\frac{q^2B^2}{8m_{W,B}^{*}}\rho ^2, \end{aligned}$$

Taking into account the ratio between Al and Ga fractions, the values of the input parameters follow from the expressions: $V_0^e=0.87x$ eV, $V_0^h=0.58x$ eV^59^, which are the potentials for electron and hole, respectively, with *x* representing the Al concentration. The potential for holes was adjusted by using the 60 : 40 offset rule. In addition, the expressions for the electron and hole effective mass as functions of the Al concentration are $m_e^*=(0.067+0.084x)\,m_0$  and $m_h^*=(0.51+0.20x)\,m_0$^60^, where $m_0$ is the free electron mass.

The Eq. (2) is numerically solved by FEM, as implemented in the Comsol-Multiphysics software^61^. Thanks to the azimuthal symmetry, the running time is considerably shorter than for a 3D equivalent model. Note that Eq. (1) is valid independently for electrons and holes. In each case, the corresponding parameters of the effective mass, the vector potential, the carrier charge and the confining energy function are taken.

On the other hand, the electron-hole ground state Coulomb interaction, in a first order approximation, can be calculated by means of the so-called Coulomb integral

5 $$\begin{aligned} C_{eh}=\frac{q^2}{4 \pi \varepsilon _0\varepsilon _r}\int _\Omega \frac{\left| \psi _e^1\left( \mathbf {r}_e \right) \right| ^2 \left| \psi _h^1 \left( \mathbf {r}_h \right) \right| ^2 \rho _e\,\rho _h }{\left| \mathbf {r}_e-\mathbf {r}_h \right| }d\rho _e\, dz_e\, d\varphi _e\, d\rho _h\, dz_h\, d\varphi _h, \end{aligned}$$

where $\varepsilon _0=8.85\times 10^{-12}$ F/m, $\varepsilon _r=13.1$. The functions $\psi _e^1$, $\psi _h^1$ are the ground states for the electron and hole, respectively, calculated from Eq. (2). The $\mathbf {r}_e(\rho _e, \varphi _e, z_e)$ is the position of the electron and $\mathbf {r}_h(\rho _h, \varphi _h, z_h)$ the hole position. Here, $\Omega$ corresponds to the six-dimensional integration space.

Another quantity that becomes useful to characterize the Coulomb interaction is the ground state overlap integral, which reads

6 $$\begin{aligned} I_{eh}=\left| \int \psi _e^1\left( \mathbf {r} \right) \psi _h^1\left( \mathbf {r} \right) \rho \,d\rho \, dz\, d\varphi \ \right| ^2\,. \end{aligned}$$

## Results and discussion

**Figure 3:**
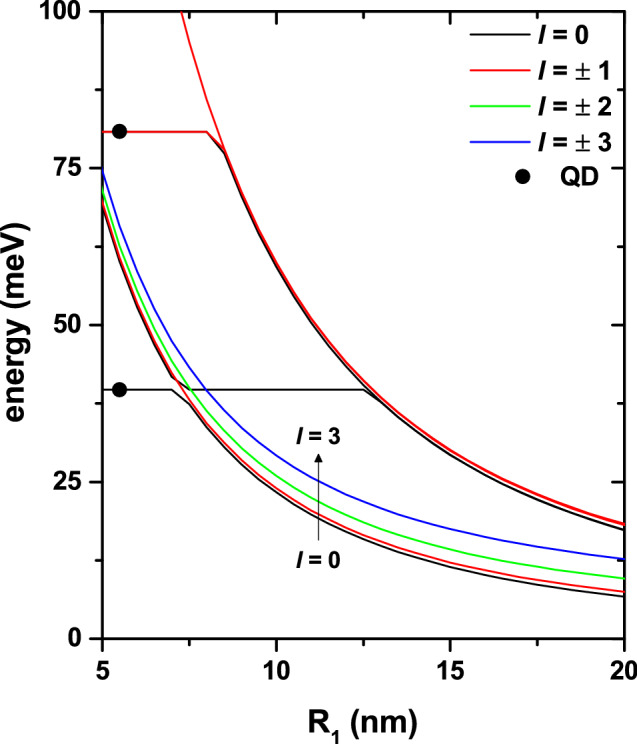
The lowest energy levels of an electron confined in a GaAs-Al$_{0.28}$Ga$_{0.72}$As coupled quantum dot-quantum ring, plotted as functions of radius $R_1$, for fixed parameters $R_2=10$ nm, $F=0$, and $B=0$. The coordinates of the ring and dot centers are (30, 35) nm and (0, 65) nm, respectively. Full symbols correspond to the case of a spherical quantum dot spectrum (with $R_2=10$ nm), without quantum ring presence. Calculations are depicted for magnetic quantum number $|l|\le 3$. Note that for $l\ne 0$, all the states are doubly degenerated.

In this section, we report on the results obtained for the energy spectrum of an electron confined in the QDR structure, taken as a function of the geometric parameters ($R_1$, $R_2$, $x_D$, $y_D$, $x_R$, $y_R$, and $\theta$), as well as of the intensity of the applied fields (*F* and *B*). We present some images with the wave functions for -uncorrelated- confined electrons and holes in the structure. Additionally, we discuss the electron-hole interaction mediated by their positions, via the Coulomb and overlap integrals. In the case of applied electric and magnetic fields, magnitudes that are within the possibilities of being experimentally implemented will be considered^58,62,63^.

The Fig. 3 shows the lowest energy levels of an electron confined in the GaAs-Al$_{0.28}$Ga$_{0.72}$As QDR as functions of the $R_1$-ring radius with $R_2=10$ nm, $F=0$, $B=0$, and keeping fixed the center of the ring and dot transversal sections (see Fig. 2a). The full symbols correspond to the levels of a spherical QD without the quantum ring presence. In the figure, two -practically not interacting- very-well defined components of the spectrum are observed; the horizontal lines correspond to the QD spectrum while the decreasing lines correspond to the energies of the ring. To verify this interpretation, we have decided to remove the QR from the structure and calculate the spectrum of the sole QD, whose radius remains constant. Clearly, as the ring has been removed, the QD spectrum must not be affected by the value of $R_1$. Note the exact match between the horizontal lines, obtained by solving the entire problem, and the isolated QD spectrum, shown by the solid symbols. Given the fixed structure dimensions that have been considered, the maximum value allowed for $R_1$, without the system collapsing due to QD-QR merge, is 32 nm. Results of the type shown in Fig. 3 correspond to values of $R_2$ up to 20 nm. So, the separation between the QR and the QD is large enough as the two systems do not interact. This fact explains the appearance of independent QD and QR spectra. The decreasing character of the spectrum of the ring appears because, when the $R_1$ value increases, there is an increment of the volume region where the electron is confined. In this figure, it is important to highlight that in the entire range of $R_1$ calculated, the ground state corresponds to $l=0$ (see Eqs. (3) and (4)). Additionally, it should be noted that, given the axial symmetry of the system, the states with $+l$ and $-l$ are degenerate. Finally, we note that the QD spectrum (horizontal lines in the total spectrum of the system and full symbols in the spectrum of the isolated QD) has a different degeneracy than that of the ring. For example, the quantum dot’s ground state is unique, while its first excited state is triply degenerate ($p_x$, $p_y$, and $p_z$ states with the same energy value).

**Figure 4:**
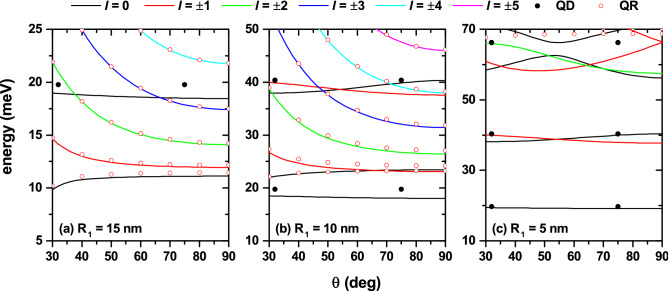
The lowest energy levels of an electron confined in a GaAs-Al$_{0.28}$Ga$_{0.72}$As coupled quantum dot-quantum ring, plotted as functions of the $\theta$-angle (see Fig. 2c) with $F=0$ and $B=0$. Calculations correspond to $R_2=15$ nm with $R_1=15$ nm (**a**), $R_1=10$ nm (**b**), and $R_1=5$ nm (**c**). The fixed position of the dot is $x_D=0$, with $z_D=65$ nm; whereas the variable ring position is given by $x_R=(R_1+R_2)\,\sin \theta$ with $z_R=z_D-(R_1+R_2)\,\cos \theta$. Note that, under these cicumstances, the quantum ring and quantum dot are tangentially in touch. The open/full symbols are for the independently determined quantum ring/dot spectra. Calculations are depicted for $|l|\le 5$. Note that for $l\ne 0$, all the states are doubly degenerated.

Figure 4 shows the lowest energy levels of an electron confined in a GaAs-Al$_{0.28}$Ga$_{0.72}$As QDR as a function of $\theta$-angle (see the Fig. 2c) with $F=0$ and $B=0$. The $\theta$-angle is varied in such a way that the QD and the QR always remain in touch. The calculations are for fixed $x_D$, $z_D$, and $R_2$ parameters with three different values of the $R_1$ radius. With the purpose of interpreting the results, the independently calculated QD and QR spectra are also shown. The QR is calculated, removing the QD, and the QD is calculated, removing the QR. The former is displayed with open symbols, while the latter is displayed with solid symbols. In Fig. 4a, where $R_1=R_2=15$ nm, there are two well-defined spectra: (i) a set of decreasing states with $\theta$ and which, in general, correspond to $l\ne 0$; (ii) one state, with $l=0$, which for $\theta \sim 30^\circ$ show an increasing character and then acquire a constant behavior; and (iii) one state that have a quasi-constant behavior, but slightly decreasing (with energy 19 meV at $\theta =32^\circ$ and with energy 18.5 meV at $\theta =75^\circ$; this state corresponds to $l=0$; see the full symbols in Fig. 4a). It can, then, be noticed that items (i) and (ii), listed above, correspond to the QR spectrum while item (iii) corresponds to the QD spectrum. Of course, the assignment is not exact since, because the two structures are in contact (see Fig. 2c), there is an interaction between the states coming from each spectrum. This means that the wave functions of those states located in the QR region have a certain penetration towards the QD region and vice versa. The generally decreasing behavior of the QR spectrum has to do with the fact that, when the $\theta$-angle increases, the torus radius increases (see $x_R$ in Fig. 2a) and, with this, it produces an increase in the volume of the toroidal region where the electron is confined, thus causing a reduction in the degree of spatial confinement. The slope of all the states shown in the Fig. 4a becomes zero when $\theta =90^\circ$. This is explained by the fact that the maximum value of the radius of the torus is obtained ($x_R$ reaches its maximum value). Since the QR and QD always remain in contact, for $90^\circ \le \theta \le 150^\circ$ a symmetric behavior must be presented to that obtained for $30^\circ \le \theta \le 90^\circ$, which accounts for the null value of the slope of all states at $\theta =90^\circ$. In Fig. 4b, where $R_1=10$ nm and $R_2=15$ nm, it is observed that the spectrum is quite similar to the case shown in Fig. 4a, but with some differences associated with the decrease in the QR cross-section. In this case, the volume of the QD becomes more relevant; in fact now the ground state of the system corresponds to electrons located in the QD region. Additionally, the spectrum associated with the QR has shifted towards higher energies, preserving, in general, its main characteristics shown in Fig. 4a. In the case of the two lowest energy levels associated with the QR, a crossover is observed around $\theta =70^\circ$, which is essentially associated with the fact that the QR ground state is much more sensitive to the decrease in the cross-section of such structure. In Fig. 4c, where $R_1=5$ nm and $R_2=15$ nm, it is observed that the ground state of the system, regardless of the $\theta$-angles, corresponds to electrons confined in the QD; this situation now extends even to the two lowest excited state; i.e., even though the two lowest excited states correspond to electrons confined in the QD with some important contribution of the wave functions penetrating the QR region. For energies greater than 50 meV, a total coupling between the two regions that make up the structure is now observed; in this case, it is impossible to assign any characteristic from the QD or the QR to the resulting energy states.

**Figure 5:**
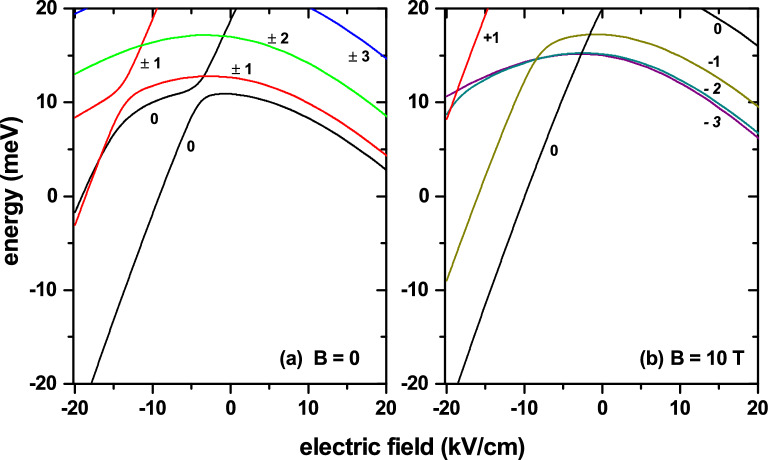
The lowest energy levels of an electron confined in a GaAs-Al$_{0.28}$Ga$_{0.72}$As coupled quantum dot-quantum ring, plotted as functions of the electric field, applied in $z-$direction. The calculations correspond to the set of fixed parameters $z_0=45$ nm, $z_D=65$ nm, $R_1=R_2=15$ nm, and $\theta =45^{\circ }$. These parameters have been chosen in such a way that the QR and the QD are in touch (see Fig. 2c). In (**a**) the results are for zero magnetic field, whereas in (**b**) are for $B=10$ T. Calculations are depicted for $|l|\le 3$. Note that in (**a**) for $l\ne 0$, all the states are doubly degenerated. In (**b**), the applied magnetic field breaks the degeneracy for $+l$ and $-l$ states, with $l\ne 0$.

In Fig. 5 appear depicted the calculated lowest energy levels of an electron confined in a GaAs-Al$_{0.28}$Ga$_{0.72}$As QDR as functions of the applied electric field strength and constant values of the magnetic field. The calculations were performed using a fixed geometrical configuration, in such a way that the QR and the QD become in touch with $\theta =45^\circ$ and $z_D=65$ nm. The radius of dot and ring transversal sections are $R_1=R_2=15$ nm and $z_0=45$ nm. The Fig. 5a contains the results for zero magnetic field, whereas in Fig. 5b they correspond to $B=10$ T. In Fig. 5a, throughout the range of calculated electric fields, the ground state is obtained with $l=0$. In the range of negative electric fields, the electron is pushed towards the QD region, with the energy showing a -linear- decreasing behavior as the electric field’s intensity increases. This fact is explained by a displacement towards lower energies of the potential well bottom, associated with the applied electric field. A similar behavior is observed for positive electric fields. In this case, the electron is pressed to be -mostly- within the QR region and, again, the decreasing energy behavior that takes place as long as the electric field magnitude increases has to do with the displacement towards a lower energy of the bottom of the potential well. For positive electric fields, the ground state presents a non-linear decreasing behavior indicating that up to 20 kV/cm, the electric field’s effects have not reached saturation. In other words, electric fields greater than 20 kV/cm still have the possibility of distorting and modifying the shape and position, respectively, of the wave function associated with the ground state in the QR. In general, the electric field ranges where the states have a linear behavior with a positive slope correspond to electron states with maximum localization in the QD region. In contrast, the energy curves with variable and negative slope are associated with states with maximum QR location. For the states with $l=\pm 2, \pm 3$, which are widely extended in the structure, the maximum of the spatial location is present within the QR for the entire range of calculated electric fields, $|F|\le 20$ kV/cm.

In Fig. 5b, where a magnetic field -with an intensity of 10 T- is applied along the axial direction, one may readily notice the breakdown of degeneracy for states with magnetic quantum number $+l$ and $-l$. Considering that a negative electric field pushes the electron towards the QD region, it is clear that for these electric field values, the ground state always corresponds to $l=0$, regardless the strength of the magnetic field. It is known that a magnetic field applied parallel to the QR symmetry direction (axial direction) generates oscillations of the ground state. This means that the ground state, for $B\ne 0$, will correspond to a certain negative value of the magnetic quantum number. The higher the applied magnetic field, the lower the value of the *l*-number corresponding to the ground state on the scale of negative values. In Fig. 5b, for $F<-2.6$ kV/cm, the ground state corresponds to $l=0$, while for $F>-2.6$ kV/cm it corresponds to $l=-3$. In general, apart from lifting degeneration, the presence of the applied magnetic field does not induce fundamental changes in the physics of the energy curves.

**Figure 6:**
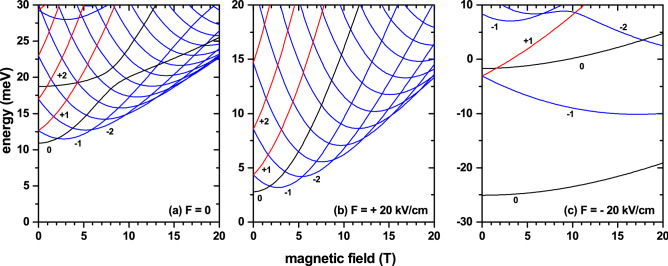
The lowest energy levels of an electron confined in a GaAs-Al$_{0.28}$Ga$_{0.72}$As coupled quantum dot-quantum ring, plotted as functions of a magnetic field, applied in $z-$direction. Three values of the applied electric field are taken into account: $F=0$ (**a**), $F=+20$ kV/cm (**b**), and $F=-20$ kV/cm (**c**). Results correspond to a fixed configuration with $z_0=45$ nm, $z_D=65$ nm, $R_1=R_2=15$ nm, and $\theta =45^{\circ }$. The parameters have been chosen in such a way that dot and ring are in touch as shown in Fig. 2c. Some values of the *l*-quantum number are indicated. Black lines are for $l=0$ and blue/red lines for negative/positive values of *l*.

Linear regions with a positive slope continue to appear and are associated with electrons in the QD region; and there are also regions of a variable slope, from positive to negative, associated with electrons in the QR.

The Fig. 6 depicts our findings for the magnetic-field dependence of the lowest energy levels in the case of an electron confined in a GaAs-Al$_{0.28}$Ga$_{0.72}$As QDR. The calculations were performed for a fixed configuration with parameters $z_0=45$ nm, $z_D=65$ nm, $R_1=R_2=15$ nm, and $\theta =45^{\circ }$. These values were chosen in such a way that the QR and the QD are in touch (see Fig. 2c). In addition, three values of the applied electric field have been taken into account. Some values of the *l*-number have been assigned to the corresponding curves as a guide to the eye. When solving the system as a whole the resulting spectrum actually comes from the interaction between the spectra of the QR and a QD. Analyzing for energies less than 30 meV in Fig. 6a, in general, the states that are doubly degenerate at $B=0$ (with $l\ne 0$) unfold into states whose energies are always increasing with the magnetic field ($l>0$, red lines), and states with energies that initially decrease and, then, for a certain value of the magnetic field increase, after reaching a minimum ($l<0$, blue lines). With the behavior of a quantum ring ($l<0$), these last states lead to oscillations of the ground state as functions of the applied magnetic field. Note that for $B=7.5$ T the ground state corresponds to $l=-2$, while for $B=4$ T the ground energy comes from $l=-1$ state. It should be borne in mind that in the ideal spectrum of a QR, the energy states with $l=0$ are always functions whose slope is monotonically increasing with the magnetic field (black lines). The two lowest energy states with $l=0$ (black lines) show the presence of QD in the system. The one with the lowest energy presents a growing slope for low fields that, later, becomes an approximately constant slope when $B>8$ T. In the case of the first excited state with $l=0$, its low-field slope is approximately zero and, from $B=8$ T, the slope of the curve systematically augments with the field. This behavior of the two states translates into a repulsion or anti-crossover between them, as shown in Fig. 6a in the region $B\sim 7$ T with energy of $\sim 20$ meV.

In Fig. 6b, containing the results for the electron energy in the case of an electric field of $+20$ kV/cm, we face the situation in which the carrier is pushed towards the region of the QR and, along the entire range of energies shown, any type of interaction with the QD disappears. It is possible to observe a number of particularities that reinforce the discussion of Fig. 6a: (i) energies of states with $l>0$ are always augmenting; (ii) state energies with $l<0$ have a mixed behavior in their slope, which is initially negative, going through zero at a certain value of magnetic field intensity (depending on the value of *l*) and, then, systematically increases until reaching a saturation value; and (iii) state energies with $l=0$ always display a monotonically increasing and positive slope until it reaches saturation.

In Fig. 6c, where an electric field of $-20$ kV/cm is applied, the electron is pushed towards the QD region. The set of the four lowest energy states (two states with $l=0$, one state with $l=-1$, and one state with $l=+1$) can be directly associated with confinement in the region of the QD, with a slight disturbance due to the presence of the QR. The following characteristics of confinement within the QD are: (i) the ground state always corresponds to $l=0$, regardless of the applied magnetic field, thus resulting in the absence of ground state oscillations; and (ii) at zero magnetic field, in the case of an isolated QD, the first excited state is doubly degenerate (two states with symmetry $2p_x$ and $2p_y$. Note that the electric field breaks the degeneracy associated to the $2p_z$ state); In the case shown in Fig. 6c, one may notice a rise in degeneration at $B=0$, which reveals the existence of the disturbance from the QR and the electric field. Additionally, Fig. 6c shows that the states with energy greater than 5 meV are associated with confinement within the QR, evidenced by the double degeneration at $B=0$, and the behavior of the slopes of the curves with $l<0$ and $l>0$.

**Figure 7:**
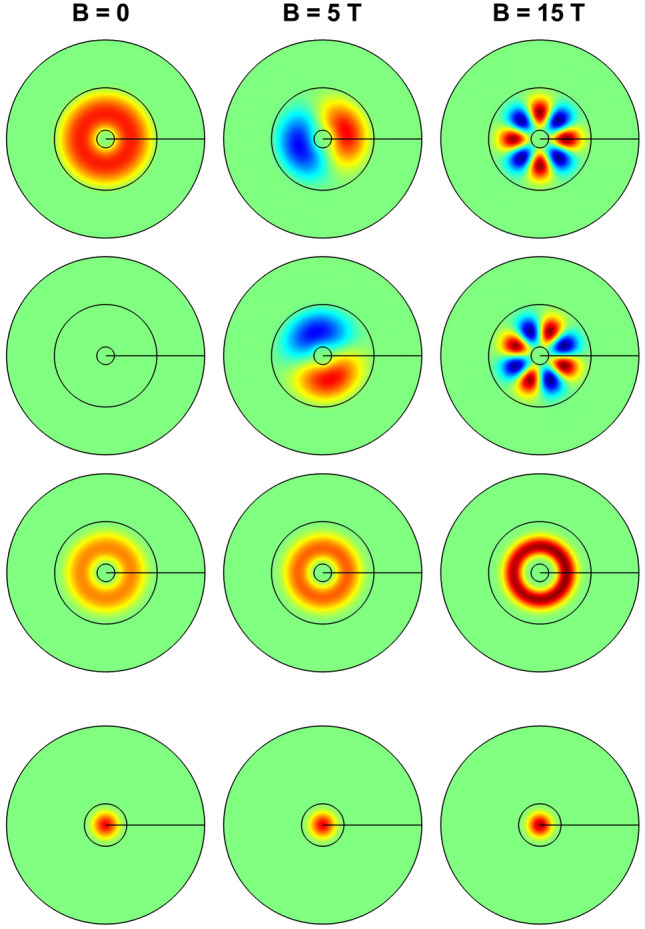
Effects of the applied electric and magnetic fields on the ground state electron wave function and probability density. The first three rows are for $F=+20$ kV/vm with $z=44$ nm, whereas the fourth row corresponds to $F=-20$ kV/vm with $z=65$ nm. Each column corresponds to a fixed magnetic field value. The first/second row is for $\mathfrak {I}{(\psi _1)}$/$\mathfrak {R}{(\psi _1)}$, whereas in the third and fourth we plot the function $\vert \psi _1\vert ^2=\mathfrak {I}{(\psi _1)}^2+\mathfrak {R}{(\psi _1)}^2$.

In Fig. 7 we have plotted the effects of the applied electric and magnetic fields on the ground state electron wave function and probability density. The first three rows show results obtained for $F=+20$ kV/cm, with the vertical carrier position set at $z=44$ nm, whereas the fourth row corresponds to the case in which $F=-20$ kV/cm, with $z=65$ nm (see Fig. 2). The calculation uses three different values of the applied magnetic field: zero (left-hand column), $B=5$ T (central column), and $B=15$ T (right-hand column). Depending on the strength of the electric field, two different planes have been chosen to project the wave functions and their corresponding probability density. In the case of positive electric field, it is confirmed that the ground state corresponds to an electron located in the QR region, whilst for the negative electric fields (fourth row), the electron is forced to locate in the QD region. The figure shows the following characteristics: (i) in the case of an electron confined to the QR region, at zero magnetic field, the ground state is a function that has no imaginary component (see the figure in the second row, first column); (ii) when the electron locates inside the QD, regardless of the value of the applied magnetic field, the ground state corresponds to $l=0$, and its wave function has no imaginary part (see the fourth row); (iii) when the electron confines within the QR, the number of nodes in the real and imaginary parts of the wave function increases as a function of the increment of the applied magnetic field intensity (see the first and second rows); this means that the value of the *l*-quantum number corresponding to the ground state augments with *B*; and (iv) the probability density has the azimuthal symmetry of the system, regardless of the applied electric and magnetic fields (see the third and fourth rows). The Fig. 7 confirms the interpretation of the results discussed in Figs. 5 and 6.

**Figure 8:**
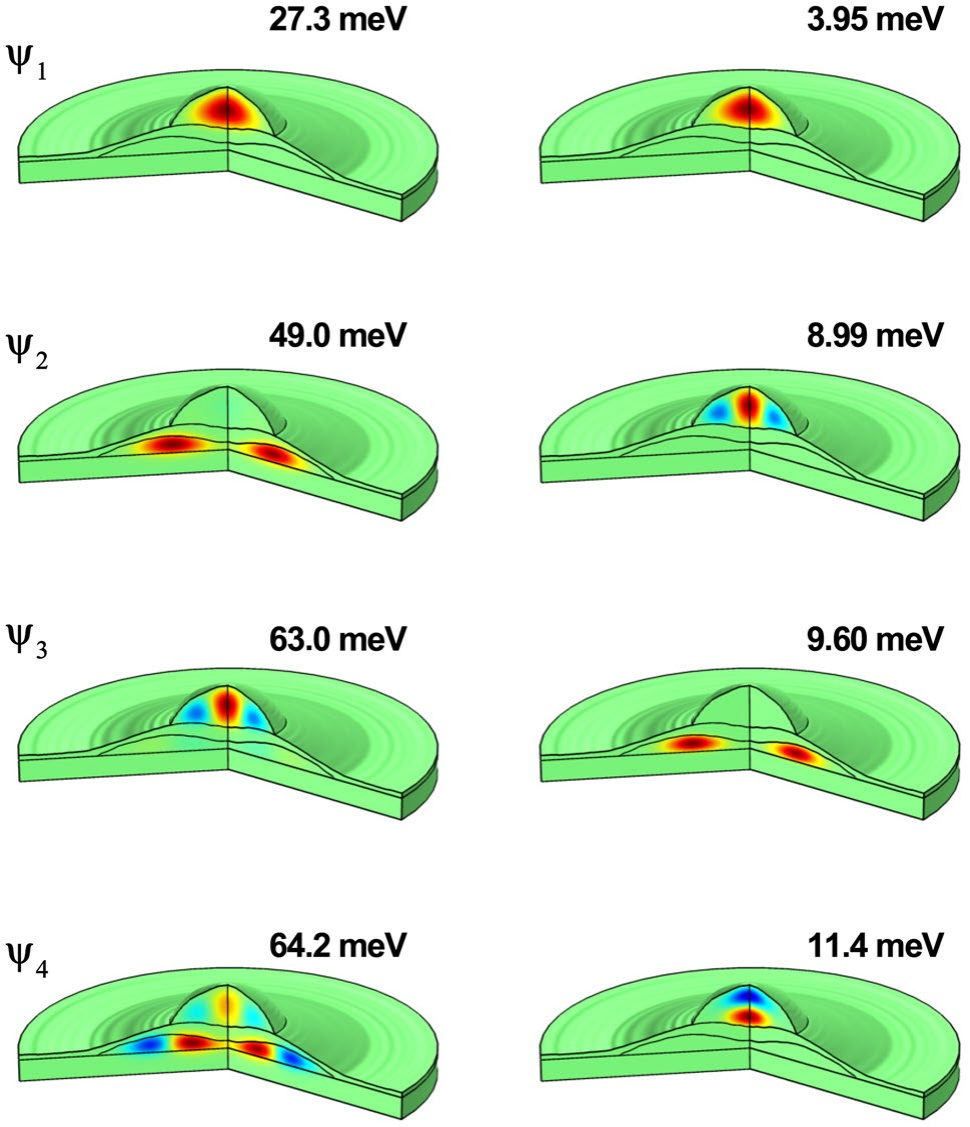
The first normalized four wave functions for two different kinds of carriers confined in a GaAs-Al$_{0.28}$Ga$_{0.72}$As in a quantum dot-quantum ring of the prototype developed in Ref.^54^: (i) the left-hand column is for electrons and (ii) the right-hand column is for holes. Above each panel, the energy value of the state is indicated.

In the remaining part of this article, we have applied our methodology to study an electron-hole pair confined in the structure obtained experimentally in the work by Elborg et al.^54^, see Fig. 1. In Fig. 8, we present the wave functions of the ground and first three excited states for an electron (left-hand column) and a hole (right-hand) confined in the QDR system described in Fig. 1a,b. In each case, the energy value of the displayed state appears indicated. Consistently with the relative size of the QD’s with regard to the QR one, it is possible to observe that the ground state ($\psi _1$), for both electron and hole, corresponds to carriers confined within the QD region. For the first excited state ($\psi _2$), it is seen that the hole remains confined inside the QD while the electron has migrated to the QR region. In the case of the second excited state ($\psi _3$), a migration of the hole towards the QR region is observed, but the electron returns to the QD region. Finally, for the third excited state ($\psi _4$), a strong localization of the hole is observed within the QD region, whereas -now- the electron wave function has a presence in both the QD and QR regions. The described coming and going of the carrier states between the QD and QR regions is fundamentally associated with the effective mass value, which translates into a greater or lesser value of the carrier’s effective Bohr radius. The wave functions of electrons, given their lower effective mass, extend mostly throughout the entire structure. In the case of holes, they are states with greater localization and, therefore, become less affected by the confinement effects associated with the band-offset in the structure.

**Figure 9:**
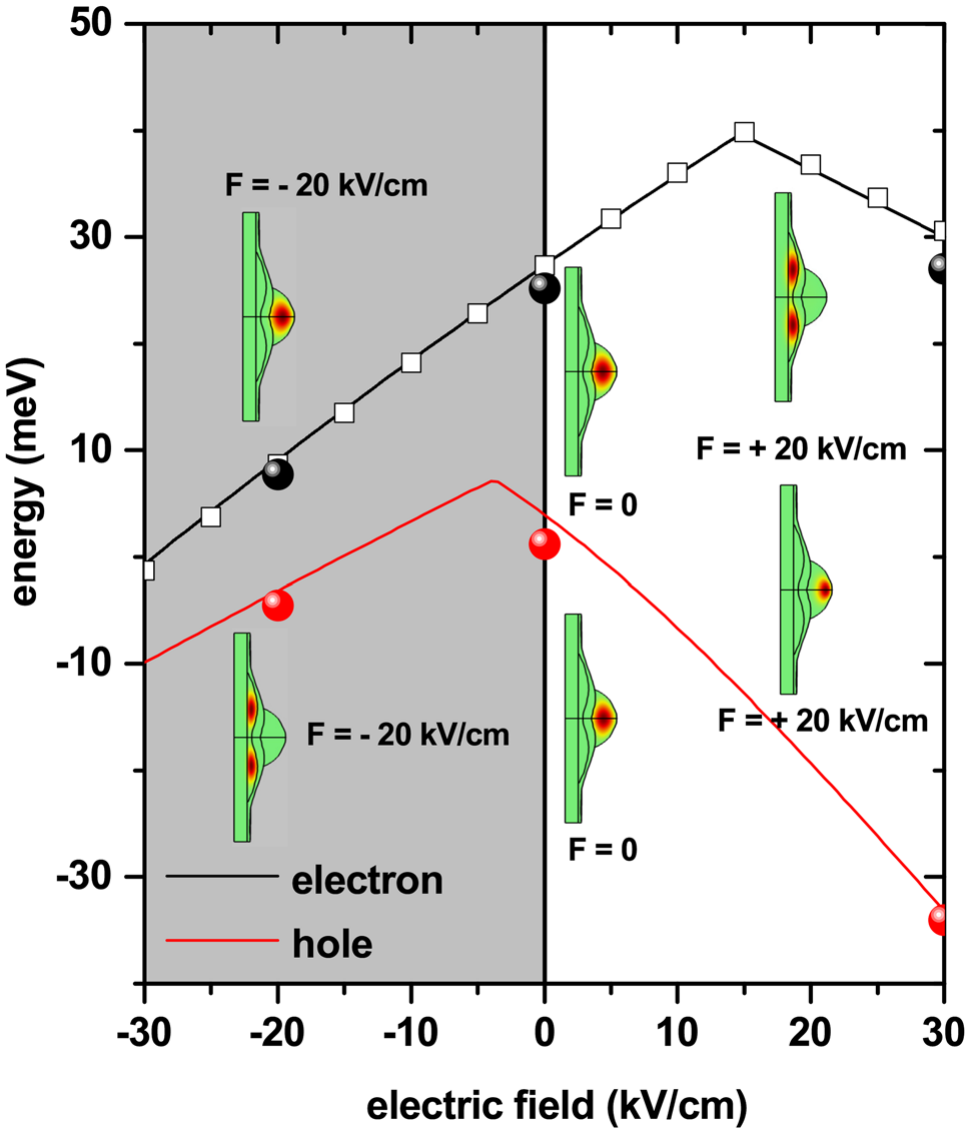
The ground state energy level for an electron ($E_1^e$) and a hole ($E_1^h$) confined in a GaAs-Al$_{0.28}$Ga$_{0.72}$As quantum dot-quantum ring under applied electric field. The distribution of wave functions for specific values of electric field is depicted. Open squares correspond to the electron ground state as a function of the applied electric field, obtained by using a diagonalization technique^64^. Full sphere symbols are the electron and hole ground states, obtained though $\mathbf {k}\cdot \mathbf {p}$ calculations^65–68^.

Continuing with the analysis of the structure obtained experimentally in^54^, Fig. 9 shows the effects of an applied electric field on the ground state energy for uncorrelated electron and hole carriers confined in the QDR coupled system ($E_1^e$ and $E_1^h$). The electric field is applied along the structure growth direction, being positive when it is directed from the annular region towards the QD with $z_0=20$ nm. From Fig. 9, it can be seen that the electric field induces a mixed behavior for $E_1^e$ and $E_1^h$: (i) while for electrons, a region with a positive slope that covers the interval $-30$ kV/cm$<F<15$ kV/cm, but in the case of the hole it occurs in the interval $-30$ kV/cm$<F<-5$ kV/cm; and (ii) a region of energy variation with a negative rate which, for the electron and the hole are within the ranges 15 kV/cm$<F<30$ kV/cm and $-5$ kV/cm$<F<30$ kV/cm, respectively. Note that the highest values of $E_1^e$ and $E_1^h$ take place for different intensities of the applied electric field.

The wave functions depicted for those three electric field values show that, for $F<0$, the electron/hole is located in the QD/QR region. On the other hand, when $F=0$, both carriers are in the QD region -which is consistent with the results in Fig. 8; and for $F>0$, the electron/hole is placed in the QR/QD region. The behavior of the curve for the electron, for example, can be interpreted as follows: At $F=0$, the electron is located in the QD region. Then, the presence of a positive electric field, up to 15 kV/cm, systematically pushes the electron towards the potential barrier that separates the QD and the QR, producing an increase in energy since the confinement effect is reinforced. Once the value of 15 kV/cm is exceeded, the electron tunnels towards the QR region and its ground state is now confined by the triangular potential associated with the potential barrier that exists at the base of the QR, which systematically moves towards lower energies as the electric field grows (this explains the decreasing character of the energy for $F>15$ kV/cm). Finally, in the range of negative electric fields, the electron is pushed towards the upper end of the QD; where, by action of the electric field, there is a displacement of the potential well bottom to lower values of the energy (there, a triangular potential with increasing field-induced depth is generated, causing a red-shift of $E_0^e$). The description of the ground state’s behavior for the electron is identical to what can be inferred for the hole, but taking into account the opposite sign of the charges. By considering the situation of a correlated electron-hole pair, it can be concluded that both positive and negative electric fields would give rise to spatially indirect excitons with the two carriers located separately in the QD and QR regions, while the spatially direct exciton appears for a null electric field with both carriers located inside the QD region.

**Figure 10:**
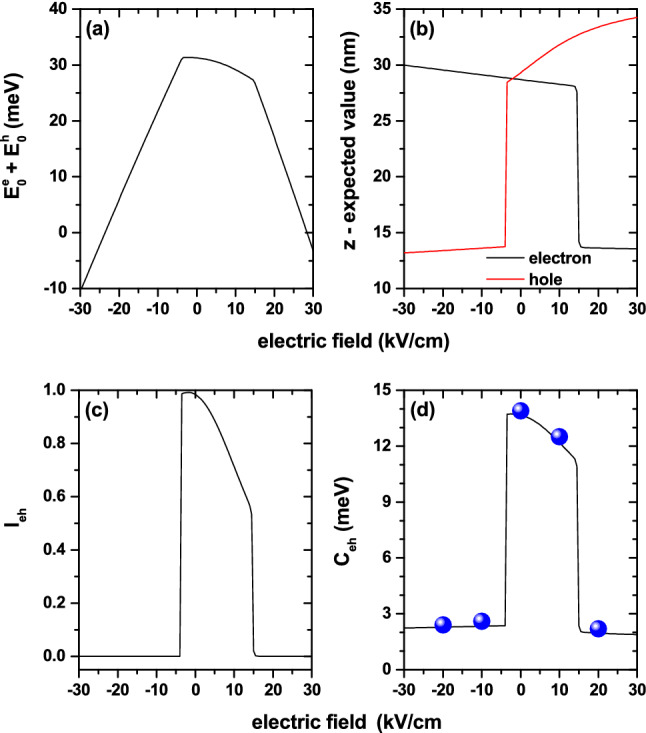
Results for electron and hole confined in a GaAs-Al$_{0.28}$Ga$_{0.72}$As quantum dot-quantum ring: the sum of ground state energy levels of electron and hole ($E_0^e+E_0^h$) (**a**), the position expected value of the electron and hole along $z-$direction (**b**), the overlap integral according Eq. (6) (**c**), and the Coulomb energy obtained via the Eq. (5) (**d**). The solid spheres in (**d**) correspond to variational calculations with a two parameters trial wave function.

We shall briefly report on the analysis of the electrostatic interaction between both carriers in the same system described in Figs. 1, 8, and 9. The Fig. 10 presents, as functions of the applied electric field, the results for the sum of the electron and hole ground state energy levels ($E_0^e+E_0^h$) (a), the expected value of the electron and hole positions along the *z*-direction (b), the electron-hole overlap integral (c), and the electron-hole Coulomb energy (d). The curve in Fig. 10a results from the direct summation of the values shown in Fig. 9. In those regions of Fig. 9, where both states show increasing or decreasing energies concerning the applied electric field, the sum in Fig. 10a is also an increasing or decreasing function with the electric field. In the range $-5$ kV/cm$<F<30$ kV/cm, where $E_0^e$ and $E_0^h$ have opposite behaviors, the resulting sum is a decreasing function of *F*. Given the greater value of the hole effective mass, its wave function is more localized. This fact allows the effects of the electric field to be more pronounced than in the case of the electron. This situation explains the reason why the resulting sum in Fig. 10a in the range $-5$ kV/cm$<F<30$ kV/cm is dominated by the $E_0^h$ behavior.

In Fig. 10b, where the results are presented for the position expected value of the carriers along the *z*-direction, it is observed that for $-30$ kV/cm$<F<-5$ kV/cm and 15 kV/cm$<F<30$ kV/cm both carriers are located in opposite regions of the structure. In the first range of the electric field values, the electron/hole is in the QD/QR region. However, in the second interval of field values that situation reverses, and the electron/hole confines in the QR/QD region. Although they are not located precisely in the same position, it can be seen from the figure that for $-5$ kV/cm$<F<15$ kV/cm, the two carriers essentially locate in the QD region. The variable slope of the curve that represents the expected value of the hole position, in the range $-5$ kV/cm$<F<30$ kV/cm, comes from the greater location of the wave function associated with the higher effective mass value.

Consistent with the results of Fig. 10b, the overlap integral is zero when the two carriers are in opposite positions in the structure (i.e., the electron in the QD and the hole in the QR, and vice versa) and is maximum when the two carriers coincide within the same space region along the *z*-direction, as shown in Fig. 10c. With the two carriers located in the QD, the overlap integral decreases systematically as the hole is pressed towards the upper region of the QD, and the electron is pressed towards the potential barrier that separates the QR and QD regions. Combining the results in Fig. 10b,c, it is then possible to understand the behavior of the electron-hole Coulomb energy, which is plotted in Fig. 10d. The Coulomb energy is minimal, but not zero, when both carriers are at opposite positions in space, in which case the overlap integral is also minimal, but not exactly zero. In those ranges of the electric field where the electron and the hole coexist in the QD region -with the integral overlap being significantly close to one-, the Coulomb energy exhibits its maximum values close to 14 meV. Note the transition between spatially direct and indirect excitons through the applied electric field effects, with changes in Coulomb energy of the order of 12 meV.

It is important to note that our results have been compared with other that arise from quite elaborate numerical schemes. In particular, to confirm the correctness of electronic and hole states as functions of the applied electric and magnetic fields, we have compared the FEM calculations with the results obtained through a process that implies the construction and subsequent diagonalization of a Hamiltonian matrix with the help an orthonormal basis composed of a product between Bessel and sinusoidal functions. The Bessel functions are used to describe the $\rho$-dependence of the wave function, whereas the sinusoidal functions are used to describe the corresponding *z*-dependence^64^

7 $$\begin{aligned} \psi (\rho ,\varphi ,z)= \sum _{m,n,l}C_{m,n,l}\,\sin \left( \frac{m\,\pi \,z}{L}+\frac{m\,\pi }{2}\right) \,J_l\left( \frac{k_{nl}\,\rho }{R}\right) \,e^{il\varphi }\,, \end{aligned}$$

where *R* and *L* are the width and height of the rectangle in Fig. 2a (with the origin of coordinates located at the half-height of the rectangle left-side in Fig. 2a), and $k_{nl}$ is the *n*-th zero of the $J_l$ first order Bessel function. The results obtained by FEM and diagonalization methods have been found to coincide within a precision of 0.1 meV. In Fig. 9, the open squares correspond to the results obtained with the diagonalization technique for the electron ground state as a function of the applied electric field; and, clearly, they match with the corresponding FEM calculations. Also, seeking to further testing the model, we have compared simulation results with a previous $\mathbf {k}\cdot \mathbf {p}$ treatment^65–68^, which considers 8 bands and configuration interaction and is solved via a plane-wave expansion. In accordance, full sphere symbols in Fig. 9 are the electron and hole ground state energies for zero and two different values of the applied electric field obtained via the mentioned $\mathbf {k}\cdot \mathbf {p}$ approach. The size of the plane-wave basis was increased until a 0.1 meV convergence was reached both for the electron and hole ground state. The calculations with the FEM and $\mathbf {k}\cdot \mathbf {p}$ method present an average difference of $7\%$, being in all cases the energies obtained by the $\mathbf {k}\cdot \mathbf {p}$ method lower than those calculated by the FEM. It is important to note that in the calculations with the $\mathbf {k}\cdot \mathbf {p}$ method the strain effects have not been included since from the beginning it has been said that the droplet epitaxy technique has, among its great advantages, the growth of strain free heterostructures. As the simulated energy levels deviate only by a few percent, we consider the present -single band- model a reasonable approximation for the discussion of more general effects.

It is also worth saying that the perturbative calculation for the correlated electron-hole pair problem was corroborated through variational calculations, including the Coulomb interaction between the two charge carriers. For this effect, two types of trial functions were used:

**Figure 11:**
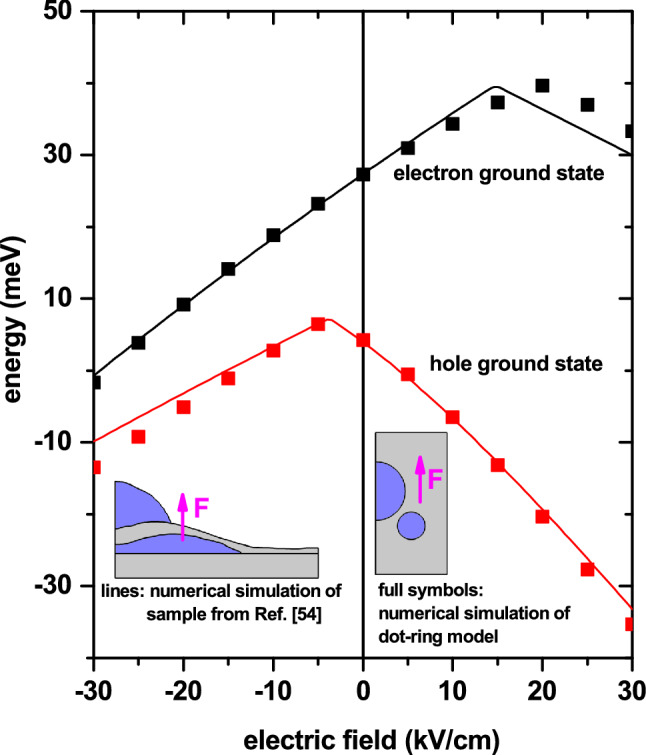
The ground state energy level for an electron and a hole confined in a GaAs-Al$_{0.28}$Ga$_{0.72}$As quantum dot-quantum ring under applied electric field. The solid lines are the calculations for the structure depicted in Figs. 1a,b whereas the full symbols correspond to the calculations for the ring-dot model shown in Fig. 1c. The dimensions of both structures are reported in Fig. 1.

(i) a hydrogenic-like function with one variational parameter^69,70^ and (ii) a trial function constructed from the product of two independent Gaussian functions. In this second case, two variational parameters were used to describe the radial problem and the problem along the axial direction separately^71,72^. Given the high computational consumption of this procedure, the test was performed for thos evalues of the electric and magnetic field for which the perturbative calculations showed a maximum in the binding energy. The results obtained by the perturbative calculation coincided with the variational ones in 97% for the binding energy. The solid spheres in Fig. 10d correspond to the expected value of the electron-hole Coulomb potential using the trial wave functions that are obtained once the energy associated with the full Hamiltonian in Eq. (1) is minimized with respect to the two variational parameters on which the trial wave functions depend^71,72^. A self-consistent calculation of excitonic states in structures with axial symmetry, such as that studied in this article, is in development and will be published elsewhere soon.

Finally, in Fig. 11, we show a comparison between the results obtained for the experimental structure grown by Elborg et al.^54^ (see Fig. 1a,b) and those corresponding to a simulated system with the simplified geometry proposed (see Fig. 1c) which, in principle, can account for the physics of the problem under consideration. In particular, we present the electron and hole ground state energy as a function of the applied electric field. The dimensions and shapes of the two structures are the same as reported in Fig. 1. The centers of the ring and dot in the simulated system are at (15, 20.5) nm and (0, 35.75) nm. Additionally, we fix $z_0=27.5$ nm. The solid lines are the same as those in Fig. 9 for the experimental structure grown by Elborg et al.^54^. The full symbols correspond to calculations related with Fig. 1c, simulating the ring-dot system. Details of numerical FEM calculations are the following: (i) for the dot-ring structure in Fig. 1c the number of elements is 17577 (triangles), the maximum/minimum element size is 3.7 nm/0.0125 nm, and the running time in an Intel Core i7 8th generation processor was 5 s; whereas (ii) for the experimental structure grown by Elborg et al.^54^ depicted in Fig. 1a,b the number of elements was 6957 (triangles), the maximum/minimum element size was 0.913 nm/0.00183 nm, and the running time with the same CPU was 3 s. The agreement is evident and allows us to conclude that the ring-dot model is a good strategy to simulate the experimental structure grown by Elborg and co-workers^54^. Of course, there are some differences, particularly for the electron case when $F>15$ kV/cm and for the hole case when $F<-15$ kV/cm, but still the physical behavior for the experimental grown and simulated system is the same. The differences essentially stem from the fact that while in the experimental sample roughly $70\%/30\%$ of the dot is surrounded by air/Al$_{0.28}$Ga$_{0.72}$As, in the dot-ring model, the dot is surrounded $100\%$ by Al$_{0.28}$Ga$_{0.72}$As. Then, in the case of the experimental sample, electrons and holes undergo the combined effects of finite and infinite potential barriers when they are in the dot region (the latter due to the presence of air), while in the simulated dot-ring system, the potential surrounding the dot, both for electrons and holes, is finite in all the space directions. Another reason that can cause the differences is associated with the elongated ring shape of the experimental sample and that, in our case, we have simplified it by using a circular cross-section. This problem could be solved by simulating the ring’s tranverse geometry with an elliptical shape, which could adapt much better to the shape of the sample from Ref.^54^.

## Conclusions

In this work, we have theoretically investigated the features of the energy spectrum of charge carriers in a GaAs-Al$_{0.28}$Ga$_{0.72}$As coupled quantum dot-quantum ring. The calculation was performed using the finite element method to solve the 3D Schrödinger-type effective mass equation in the conduction and valence bands of the system. The process takes into account, explicitly, influence of the presence of externally applied electric and magnetic fields. Accordingly, we perform a discussion about the modifications in the energy states due to changes in the geometrical setup of the dot-ring structure as well as to the applied field effects. The motivation for this research comes from a recent report on the practical realization of a in a GaAs-Al$_{0.28}$Ga$_{0.72}$As coupled quantum dot-quantum ring^54^. Hence, a part of the work relates with a model for the electron an hole states in the same kind of system. In this context, we present the calculated electron-hole energies and predict the formation of indirect excitons in the case of the application of a static electric field oriented along the growth direction, with carriers separately confining within the quantum dot and quantum well regions. A quantitative characterization of the Coulombic electron-hole interaction, via the corresponding Coulomb integral is also given. The kind of nanostructure studied in the article could be of interest for the fabrication of optoelectronic devices, with a particular possibility of exhibiting, as mentioned in the above referred work, an efficient optical Aharonov–Bohm effect.

Summarizing, we made calculations for the structure and behavior of the energy level spectrum in quantum dot-ring system as the one fabricated by Elborg et al.^54^. Up to our knowledge, ours is the first theoretical approximation to such a structure. The corresponding Schrödinger equation was solved by FEM in the effective mass approximation. As a model which can be easily interpreted we selected the spherical QD-QR system, and the most of the results correspond to this description. Then, due to the absence of further experimental results, we have checked our approach by extending the calculations, for some particular cases, to using other techniques: the matrix diagonalization method, $\mathbf {k}\cdot \mathbf {p}$-method, and the variational description. All the techniques show very similar results. This would constitute a proof for correctness of all the obtained results within the spherical QDR scheme: but then, again, we have compared some results for this model with analogous ones obtained for the real structure^54^. Such comparison evidences to correctness of the choice of a spherical QDR structure for modeling the real one.
